# RINGs, DUBs and Abnormal Brain Growth—Histone H2A Ubiquitination in Brain Development and Disease

**DOI:** 10.3390/epigenomes6040042

**Published:** 2022-12-02

**Authors:** Lucy Anne Doyle, Firuze Unlu Bektas, Eleftheria Chatzantonaki, Charlotte Repton, Alexandra Derrien, Robert Scott Illingworth

**Affiliations:** Centre for Regenerative Medicine, Institute for Regeneration and Repair, The University of Edinburgh, Edinburgh BioQuarter, 5 Little France Drive, Edinburgh EH16 4UU, UK

**Keywords:** chromatin, epigenetics, neurodevelopment, neurodevelopmental disorders, epigenetics, polycomb, histone modifications, H2AK119ub1, chromosomal architecture

## Abstract

During mammalian neurodevelopment, signaling pathways converge upon transcription factors (TFs) to establish appropriate gene expression programmes leading to the production of distinct neural and glial cell types. This process is partially regulated by the dynamic modulation of chromatin states by epigenetic systems, including the polycomb group (PcG) family of co-repressors. PcG proteins form multi-subunit assemblies that sub-divide into distinct, yet functionally related families. Polycomb repressive complexes 1 and 2 (PRC1 and 2) modify the chemical properties of chromatin by covalently modifying histone tails via H2A ubiquitination (H2AK119ub1) and H3 methylation, respectively. In contrast to the PRCs, the Polycomb repressive deubiquitinase (PR-DUB) complex removes H2AK119ub1 from chromatin through the action of the C-terminal hydrolase BAP1. Genetic screening has identified several PcG mutations that are causally associated with a range of congenital neuropathologies associated with both localised and/or systemic growth abnormalities. As PRC1 and PR-DUB hold opposing functions to control H2AK119ub1 levels across the genome, it is plausible that such neurodevelopmental disorders arise through a common mechanism. In this review, we will focus on advancements regarding the composition and opposing molecular functions of mammalian PRC1 and PR-DUB, and explore how their dysfunction contributes to the emergence of neurodevelopmental disorders.

## 1. Introduction

During mammalian development, signaling pathways act in concert with tissue-specific transcription factors (TFs) to establish appropriate gene expression patterns. Transcription does not, however, occur on naked DNA, but instead in the context of the nucleoprotein complex chromatin; DNA wrapped around basic histone proteins. This configuration provides a scaffold to package the genome into the nucleus and a substrate for a plethora of posttranslational modifications. In addition, chromatin can undergo changes to its packaging state across orders of magnitude—from local regulatory domains to interactions that span entire chromosomes. These chemical and physical modifications can be permissive for, or refractory to the molecular processes that operate on DNA. Indeed this ‘epigenetic’ information ensures the appropriate and proportionate temporal and spatial transcriptional response during cellular specification [1,2,3]. In so doing, context-specific chromatin landscapes ensure the appropriate balance between stemness and lineage commitment to control growth and cellular specialisation during mammalian development.

**Figure 1:**
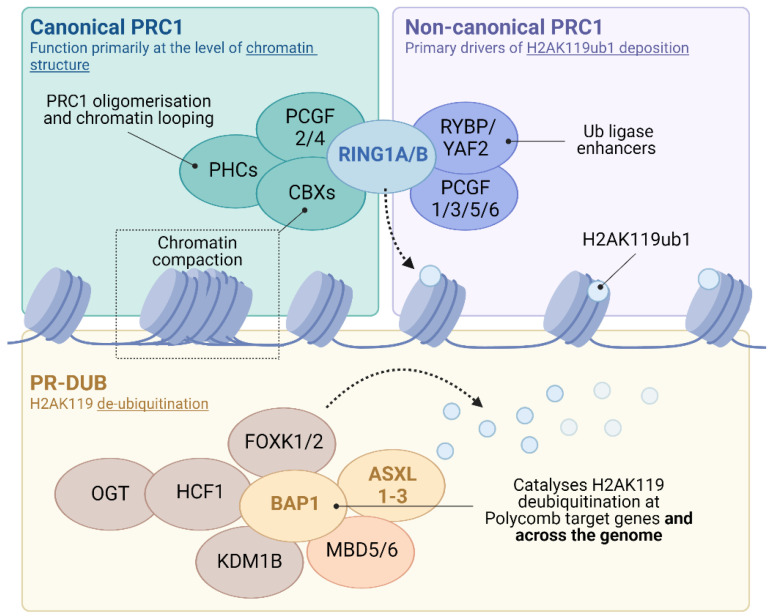
PRC1 complexes contain a highly conserved catalytic core consisting of one of two interchangeable E3 ubiquitin ligases (RING1A or RING1B), which form mutually exclusive heterodimers with one of six Polycomb group RING-finger domain proteins (PCGF1-6). Additional subunits allow the classification of PRC1 into cPRC1 and ncPRC1 forms. cPRC1 complexes assemble around a dimer of RING1A/B and either PCGF2 or PCGF4. Additional subunits including CBX2, CBX4, CBX6, CBX7 or CBX8 and PHC1, PHC2 or PHC3 proteins. CBX proteins have an affinity for histone H3 lysine methylation (e.g. H3K27me3) and are important for PRC1 targeting to chromatin alongside roles in chromatin compaction. In addition, the presence of PHC proteins enable cPRC1 to modulate higher order chromatin structure. In contrast, ncPRC1 complexes comprise dimers of RING1A/B and PCGF1, 3, 5 or 6 associated with either RING and YY1 Binding Protein (RYBP) or its homolog YY1 Associated Factor 2 (YAF2) in place of CBX. The inclusion of RYBP or YAF2 in ncPRC1 greatly enhances RING1A/B catalytic activity, such that ncPRC1s contribute to the majority of H2AK119ub1 in vivo. PR-DUB complexes contain a catalytic core of BAP1, which forms mutually exclusive complexes with one of three additional sex combs-like paralogues (ASXL1-3). PR-DUB complexes function in the cleavage of ubiquitin conjugates from both chromatin and soluble protein targets, including H2AK119ub1. Additional subunit interactions modulate PR-DUB targeting and function, including the transcription factors FOXK1/2, chromatin modifiers OGT and KDM1B, transcriptional cofactor HCF-1 and members of methyl-CpG-binding family MBD5 and 6.

One of the best characterised of the epigenetic regulatory systems is that of the polycomb group (PcG) family of co-repressors. PcG proteins assemble into multi-subunit complexes that alter both the chemical and structural properties of chromatin to antagonise transcription. These complexes sub-divide into distinct functionally related families. Polycomb repressive complexes 1 and 2 (PRC1 and 2) modify the chemical properties of chromatin by covalently modifying histone tails. PRC2 contains EZH1 or 2, SET domain methyltransferases which can mono-, di- and tri-methylate lysine 27 of histone H3 (H3K27me1-3) [4,5,6,7]. PRC1 contains the core catalytic E3 ubiquitin ligases RING1A or B, which mono-ubiquitylates lysine 119 of histone H2A (H2AK119ub1; Figure 1) [8,9]. These histone modifications support reciprocal PRC targeting, providing a self-reinforcing recruitment mechanism onto chromatin [4,10,11,12,13,14]. In addition to covalent modification, a subset of PRC1 (‘canonical’ or cPRC1) alters both local and distal chromatin architecture (Figure 1; reviewed in [15]). In contrast to the PRCs, the polycomb repressive deubiquitinase (PR-DUB) complex removes H2AK119ub1 from chromatin through the action of the C-terminal hydrolase BAP1 (Figure 1).

In mice, loss of core PcG proteins leads to early embryonic lethality with failure occurring at, or shortly after gastrulation [16,17,18]. In contrast, loss of secondary subunits leads to more subtle developmental defects that include homeotic transformations and abnormal axial body patterning [10,19,20,21,22]. Consistent with these experimental observations, genetic screening has identified PcG mutations as being causally associated with a range of pathologies, including both malignant cancers and congenital developmental disorders. Disruption of PRC1 and PR-DUB lead to neurodevelopmental disorders (NDDs) associated with both localised and/or systemic growth abnormalities. These complexes operate together to control the balance of H2AK119ub1 in the genome, and as such, it is tempting to postulate that these NDDs arise through a common mechanism. In this review, we will discuss the molecular functions of PRC1 and PR-DUB in mammals and explore how their dysfunction leads to disordered brain development.

## 2. PRC1 Composition, Targeting and Functional Compartmentalisation

PRC1 complexes contain a highly conserved catalytic core [23]. In mammals, this consists of one of two interchangeable E3 ubiquitin ligases (RING1A or RING1B), which form mutually exclusive heterodimers with one of six Polycomb group RING-finger domain proteins (PCGF1–6) [24,25,26]. The resulting PRC1 sub-complexes are denoted as PRC1.1–6 to indicate the inclusion of PCGF1–6 respectively. Biochemical studies have identified additional PcG subunits that allow the classification of these PRC1s into cPRC1 (PRC1.2 and 1.4) and ncPRC1 (PRC1.1, 1.3, 1.5 and 1.6) forms with distinct chromatin-targeting and regulatory functions [24,27,28].

### 2.1. Canonical PRC1 and the Importance of Chromosomal Folding

**Figure 2:**
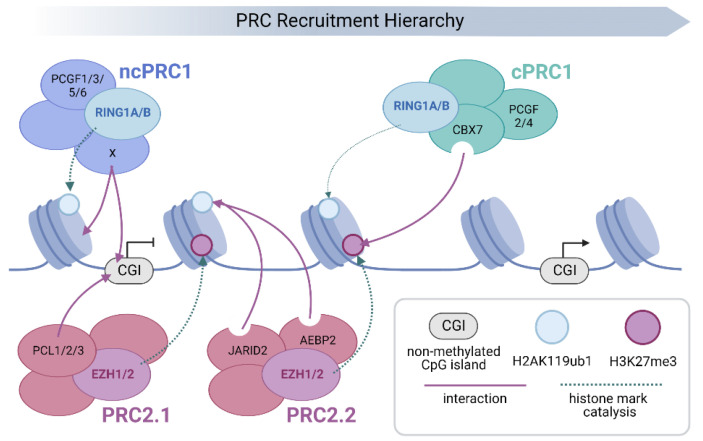
Schematic representation of Polycomb group (PcG) protein recruitment and chromatin binding. The PRC family is targeted to chromatin by a hierarchical process. Initial localisation occurs through sequence specific and/or direct recruitment mechanisms that in turn lead to the deposition of chromatin modifications that act to recruit or activate downstream PRCs. Transcriptionally silent, non-methylated CpG islands (CGIs), provide a direct recruitment platform for both PRC2.1 and PRC1.1 via affinity of their Polycomb-like (PCL; PCL1-3) and Lysine Demethylase 2B (KDM2B) subunits respectively [10,19,63,64,65,66]. In addition, other ncPRCs employ both sequence specific and RNA-mediated mechanisms to target specific sites in the genome (the varying subunits responsible for chromatin targeting of individual ncPRC1s, including KDM2B, are denoted as X for simplicity) [67,68,69,70,71,72]. This leads to the deposition of H3K27me3 by EZH1/2 (PRC2.1) and H2AK119ub1 by RING1A/B (ncPRC1). The resulting H2A ubiquitination leads to the recruitment and allosteric activation of PRC2.2, through association with JARID2 and AEBP2, which enhances H3K27me3 deposition [12,73,74,75]. Finally, cPRC1 is recruited to chromatin by CBX7-mediated recognition of PRC2-deposited H3K27me3, where it nucleates physical interactions between other cPRC1 target sites in the genome [22,29,31,37,38,42]. However, due to its very low E3 ubiquitin ligase activity, cPRC1 contributes little to the deposition of H2AK119ub1 (denoted by a faint dashed arrow), which is primarily carried out by ncPRC1s [54,55].

In mammals, cPRC1 complexes assemble around a dimer of RING1A/B and either PCGF2/MEL18 or PCGF4/BMI1. This assembly associates with additional subunits, including homologs of the *Drosophila* polycomb (Pc; CBX2, CBX4, CBX6, CBX7 or CBX8) and polyhomeotic (Ph; PHC1, PHC2 or PHC3) proteins. The chromodomain of CBX subunits has affinity for histone H3 lysine methylations (H3K27me3 and H3K9me3) and these have been shown to be important for PRC1 targeting to chromatin [29,30,31]. CBX7, the primary PRC1 chromobox paralog in mouse embryonic stem cells (mESCs) has selective affinity for H3K27me3 and, therefore, helps to target cPRC1 to sites of PRC2 occupancy [4,6,24,27,29]. CBX7-mediated recognition of PRC2-depositied H3K27me3 is, therefore, central to the hierarchical recruitment of PRCs to target genes [14,32] (Figure 2). In addition to targeting, the positively charged intrinsically disordered region (IDR) of CBX2 drives local chromatin compaction and facilitates electrostatic interactions that promote the formation of nuclear condensates [33,34,35]. In contrast, CBX4, 6, 7 and 8 lack the IDR domain and, therefore, do not contribute to chromatin compaction or phase separation [33,35]. CBX2 mediated condensates form membraneless nuclear organelles, through liquid–liquid phase separation (LLPS), and occur in vitro, even in the absence of other PRC1 subunits [33,34]. The regulatory significance of CBX2-mediated compaction and phase separation is unclear; however, mutations within the IDR give rise to abnormal axial patterning in mice [22,33,35].

Microscopic and chromosomal conformation capture (‘C’) techniques have revealed that distal chromosomal regions, separated by great genomic distances, cluster within the nucleus in a PRC1-dependent manner. Disruption of RING1B or subunits of cPRC1 lead to the loss of interactions and marked alterations to nuclear organisation [36,37,38,39]. PHC proteins (PHC1-3), obligate components of cPRC1, facilitate these long-range chromosomal contacts through head-to-tail interactions between their sterile α motif (SAM) domains [24,37,39,40,41,42,43,44]. In both flies and mammals, these distal interactions form microscopically visible nuclear foci, termed ‘polycomb bodies’, with high local concentrations of PcG proteins and characteristics of LLPS condensates [39,45,46,47]. Polycomb bodies are distinct from the short-range CBX-mediated chromatin compaction and their formation relies on highly dynamic interactions between the PHC subunits of cPRC1 complexes [31,48,49,50].

cPRC1 exhibits relatively low E3 ubiquitin ligase activity in vitro and contributes minimally to the deposition of H2AK119ub1 in vivo, suggesting that this may not be its primary molecular function [24,51,52,53,54,55,56]. This suggests that cPRC1 principally operates to alter chromosomal topology to regulate developmental gene expression patterns. Consistent with this, PHC knockout or mutations in the SAM domain disrupt both local and distal interactions, disrupt polycomb foci in the nucleus and up-regulate PRC1 targeted genes in vivo [37,39,42]. Disruption of cPRC1 through simultaneous loss of PCGF2 and 4 leads to characteristic homeotic defects in mice but only modest gene misregulation in mESCs [19,54]. This suggests that the functional contribution of cPRC1-mediated contacts is somewhat modest and manifests only as cells transition between states, either through a failure to maintain or fine tune-developmental gene expression programmes. Indeed, it has been suggested that PRCs can facilitate gene induction during differentiation by bringing poised enhancers into proximity with their target genes to allow subsequent activation [57,58,59,60,61], although recent work has cast doubt on this model [62]. A synthetic recruitment strategy highlighted the importance of cPRC1 specifically in the heritability of PRC1 binding across cell divisions [32]. Whilst the exact contribution of cPRC1 to developmental gene regulation remains unclear, repression is likely reliant on its ability to both stabilise PRC1 recruitment onto chromatin and to nucleate chromosomal interactions between target sites in the genome [37,42,54].

### 2.2. Non-Canonical PRC1 and the Deposition and Function of H2AK119ub1

ncPRC1 complexes comprise dimers of RING1A/B and PCGF1, 3, 5 or 6, associated with either RYBP or its homolog YAF2 in place of CBX [24,76] (Figure 1). The inclusion of RYBP or YAF2 in ncPRC1 has been shown to greatly enhance RING1A/B catalytic activity, contributing to the majority of H2AK119ub1 in vivo [24,55]. Deletion of all four non-canonical PCGFs results in a global reduction in H2AK119ub1 in mESCs despite the presence of intact cPRC1 [54]. This suggests that ncPRC1 complexes play a major role in shaping the level and distribution of H2AK119ub1.

The lack of CBX subunits means that ncPRC1 complexes do not recognise H3K27me3 and therefore deposit H2A ubiquitination in a PRC2-independent manner [56]. In addition PRC2, through association with JARID2 and AEBP2, can be targeted to sites of H2AK119ub1 to facilitate H3K27me3 deposition [10,11,12,73,74]. The KDM2B subunit targets ncPRC1 to non-methylated CGIs, a chromatin landmark characteristic of the majority of mammalian gene promoters [63,77]. These findings demonstrate the importance of ncPRC1-mediated ubiquitination for robust recruitment of PRCs on to chromatin (Figure 2).

H2AK119ub1 is highly abundant in mammals, with approximately 10% of total H2A in mESCs carrying the modification [49,54]. However, PRC1 function, at least as measured by gene repression and developmental competence, is robust to substantial reductions in these levels. Mouse ESCs that express catalytically hypomorphic RING1B (*Ring1b^I53A/I53A^*) present with substantially reduced global H2AK119ub1 levels (10–35% of wild-type; [78,79] and unpublished observations), but preserve near wild-type genome-architecture, cell-cycling dynamics and PRC1-mediated gene repression [36,37,79,80]. Hypomorphic RING1B mice (*Ring1b^I53A/I53A^*) die perinatally, however, as for *Drosophila* bearing a functionally equivalent mutation in the RING1B homolog Sce (*Sce^I48A/I48A^*), they do not present with the earlier embryonic developmental deficits associated with RING1B deficiency [79,81]. Global reduction of H2AK119ub1 is not homogeneous, however, and many PRC1 targets retain appreciable levels of ubiquitination that could explain the somewhat modest gene misregulation observed in *Ring1b^I53A/I53A^* cells [75,78,79]. In contrast, mESCs with more substantially impaired PRC1 catalysis (*Ring1b^I53A/D56K^* or *Ring1b^I53S/I53S^Ring1a^−/−^*) present with a more complete loss of H2AK119ub1 and leads to substantial gene misregulation [54,75,78]. These findings suggest that that there is a critical threshold of H2AK119ub1 below which the ability to repress genes is compromised. Interestingly, during skin development in mice, reduced RING1B catalysis (*Ring1b^I53A/I53A^*) does not impair gross epidermal development but does lead to ectopic expansion of non-skin cell types [52]. This suggests that the threshold of and/or the sensitivity to H2AK119ub1 misregulation may vary depending on the developmental context.

Recent observations suggest that PRC1 reduces gene expression through alterations in transcriptional burst frequency, however, the biophysical contribution of H2AK119ub1 to this is currently unknown [82]. H2AK119ub1 could act directly to regulate transcription or instead by ensuring robust placement and inheritance of the polycomb system. Interestingly, *Drosophila* embryos carrying mutations in H2A or H2Av that block PRC1-mediated ubiquitination (*H2A^K118R^* and *H2Av^K120R/K121R^* respectively) and modestly reduce H3K27me3 can still support normal embryonic development [81]. The relatively modest impact in flies suggests that mammals may have a higher requirement for H2AK119ub1. Indeed, PRC recruitment in *Drosophila* operates through a well-defined DNA sequence-dependent recruitment mechanism, yet no equivalent system has been identified in mammals (reviewed in [83]). The expansion of the mammalian genome (~0.14 Gb and 3.05 Gb for *Drosophila* and human respectively) may therefore have led to an increased dependence upon H2AK119ub1 for robust targeting of polycomb complexes in the absence of sequence specific recruitment. Further work is required to disentangle the direct versus indirect contribution of H2AK119ub1 in gene repression in mammals.

## 3. PR-DUB and the Curious Case of the Polycomb Complex That Activates Genes

The PR-DUB complex cleaves ubiquitin conjugates from both chromatin and soluble protein targets, but is best characterised for its erasure of H2AK119ub1 (H2AK118ub1 in *Drosophila*). In flies, the functional core of PR-DUB comprises the C-terminal hydrolase Calypso and its obligate partner Additional Sex Combs (Asx), that together constitute the active enzymatic core of the complex [84]. As for the PRCs, PR-DUB is conserved in mammals but has undergone expansion, with BAP1 (the mammalian orthologue of Calypso) forming mutually exclusive complexes with one of three Asx paralogues (Additional sex combs-like; ASXL1-3) [25,27,85]. These interactions are crucial to both the function and stability of mammalian PR-DUB [85,86]. Quantitative mass spectrometry demonstrates that BAP1 and the ASXL component exist with a 2:1 stoichiometry, indicating that BAP1 may be dimeric in the PR-DUB complex [27]. In addition, PR-DUB associates with subunits that modulate its targeting and function, including the transcription factors FOXK1/2; chromatin modifiers OGT and KDM1B; transcriptional cofactor HCF-1 and members of methyl-CpG-binding family MBD5 and 6 [25,87,88,89,90,91,92,93,94,95]. Although the fundamental composition of PR-DUBs is grossly similar, KDM1B has been shown to be specific to the ASXL2-containing variant [86]. Furthermore, expression of the ASXLs varies in different cell types suggesting that PR-DUB composition may show as yet unappreciated variability in a context-dependent manner [27,96].

**Figure 3:**
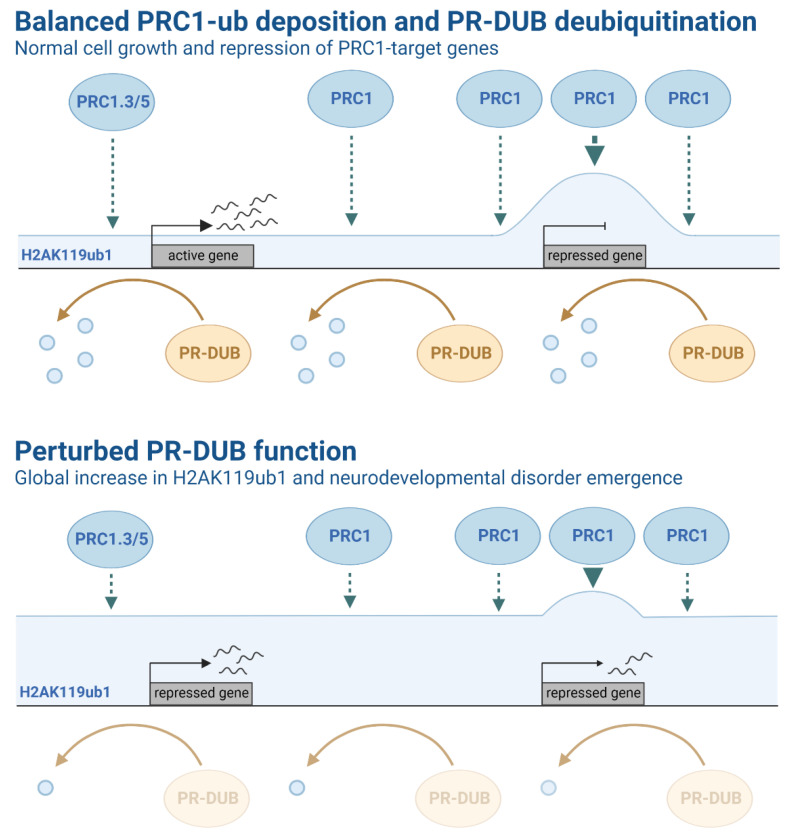
Schematic representation of Polycomb group (PcG) complex activity and related histone ubiquitination levels. Top: Active (PRC1-low, H2AK119ub1-low) and repressed (PRC1-high, H2AK119ub1-high) gene promoter regions are represented in the context of balanced PRC1-ub deposition and PR-DUB deubiquitination function. PRC1 is highly localised to target gene promoter regions, ultimately resulting in enriched levels of H2AK119ub1 and the repression of key PcG target genes. PR-DUB regulates H2A ubiquitination, which may function to maintain balanced H2A ubiquitination levels across the genome, counteracting PRC1-mediated H2A ubiquitination and leading to transcription. Bottom: Mutations in several components that modulate H2A ubiquitination have been associated with several syndromes and brain pathologies. For example, germline mutations of ASXL1-3 subunits in PR-DUB have been identified in patients with rare congenital disorders. Notably, patients with Bainbridge-Ropers syndrome have been shown to exhibit higher levels of H2A ubiquitination and differential gene expression, suggesting disruption of PR-DUB function. Two promoter regions (first: PRC1-low, H2AK119ub1-high; second PRC1-high, H2AK119ub1-high) genes are represented in the context of perturbed PR-DUB function. Reduced PR-DUB deubiquitination function results in a global increase of H2AK119ub1 at target genes.

As described above, PcG family genes are characterised as gene repressors. In keeping with this, the *Drosophila* PR-DUB subunits Calypso and Asx are classified as members of the PcG family based on the characteristic homeotic transformation and gene misregulation observed in loss-of-function screens [97,98,99]. However, that PR-DUB action counteracts the molecular activity of PRC1 appears to be at odds with these complexes acting together to repress transcription [86,100]. In support of this, the primary role of PR-DUB appears to be to promote gene activation and/or establish a transcriptionally permissive chromatin state [84,85,86,101,102,103,104]. Recent findings that PR-DUB acts to constrain pervasive genome-wide H2AK119ub1 go some way to reconciling these two, seemingly contradictory, observations. In the absence of BAP1, levels of H2AK119ub1 increase two-fold throughout the genome, leading to marked gene repression and the simultaneous activation of a small but conspicuous subset of polycomb target genes [103,105]. These observations suggest a model whereby global increases in H2AK119ub1 lead to a redistribution of a limited pool of PRCs, in turn causing inappropriate gene repression at ectopic sites and sequestering PRCs away from target genes [103,105]. This places the function of PR-DUB as a buffering mechanism against the potentially deleterious effects of excessive PRC1 activity, most notably from PRC1.3 and 5 [54,105], which could otherwise lead to genome-wide perturbation in chromatin-mediated gene regulation (Figure 3). Such balance wrought from simultaneous deposition and erasure mechanisms highlights the fine-tuning of gene expression and the potential for perturbations leading to derailed development and disease.

## 4. Polycomb Complexes and the Fidelity of Cortical Development

Neurons and glial cell types of the developing cortex derive from multipotent neural stem cells (NSCs) that pass through sequential, spatiotemporally restricted phases of neural precursor cell (NPC) expansion, neurogenesis and gliogenesis. Accumulating research highlights the importance of epigenetic regulation in this process, with PcG-mediated transcriptional regulation in particular being critical to ensure the fidelity of cortical growth and specification. In mice, targeted ablation of EZH2 prior to the onset of neurogenesis leads to an accelerated neurogenic phase, a premature switch into gliogenesis and a smaller cortex at birth [106]. In contrast, depletion of either EZH2 or RING1B in ex vivo cultured neural progenitors from later developmental stages prolongs the neurogenic phase due to a failure to silence pro-neural gene Neurog1 and leads to the delayed onset of gliogenesis [107]. Interestingly, separation of function studies have shown that different modes of PRC1 action are required as cortical development proceeds. PRC1-mediated H2AK119ub1 deposition is required for gene silencing during the neurogenic phase whereas a ubiquitination-independent mechanism is required for persistent repression of neurogenic genes to support the neurogenic-to-astrogliogenic switch [108]. The PRC1-mediated silencing of neurogenic genes is also dependent on PHC2, supporting the notion that a structural function of canonical PRC1 may contribute to this ‘maintenance’ function, although the molecular mechanism for this remains unknown [108]. Interestingly, loss of EZH2 in the midbrain leads to up-regulation of a forebrain transcriptional programme that includes the master regulators *Pax6* and *Foxg1* [109]. Finally, PRC1 activity is essential for sustaining motor neuron subtype integrity in mouse [110]. These findings demonstrate that polycomb complexes are required for the expansion of neuroepethelial cells into neurogenic radial glial cells; the specification capacity of progenitor cells to different cortical laminar fates; the timing of the neurogenic to gliogenic switch; and the correct spatio-temporal gene regulation at a gross-scale across the developing brain. Consistent with these experimental observations, mutations in subunits of all three polycomb complexes give rise to NDDs that present with intellectual disability (ID), a high incidence of severe autism spectrum disorder (ASD) and brain growth abnormalities.

## 5. NDDs of Perturbed PRC1 Function

Congenital defects in various PRC1 subunits leads to developmental deficits reflecting their role in a range of disparate developmental processes. A subset of PRC1 subunits are either upregulated during neural development and/or have context-specific functions in the developing nervous system. Perturbations in these components leads to defined NDDs, the characteristics and aetiologies of which will be explored further below.

### 5.1. RING1A

RING1A is one of two E3-ubiquitin ligases that constitute the core enzymatic subunit of PRC1. Whilst the role of the paralogous RING1B subunit is well documented for its involvement in NPC fate transitions, the role of RING1A is less clear [107,108,111]. Disruption or overexpression of RING1A in mice results in skeletal patterning defects despite unaffected *Ring1B* expression [20]. In contrast, *Ring1B* knockout results in early embryonic lethality, presumably due to RING1B’s more ubiquitous expression compared to RING1A, which is primarily detected in the central nervous system [18,20]. Recent research implicates a role for both RING1 paralogues in the regulation of spatial morphogen expression patterns crucial for mouse telencephalon ventralization and formation of the neocortex, although RING1B is thought to be the dominant contributor [112].

A heterozygous de novo mutation in RING1A (p.R95Q) was identified in a child who displayed characteristics of perturbed neurodevelopment and microcephaly in early childhood. The individual presented with delayed acquisition of language and adaptive social skills, ID and psychotic symptoms that developed at adolescence [113]. Modelling this mutation in vitro demonstrated an impaired capacity of RING1A^R95Q^ to ubiquitinate histone H2A, consistent with significantly reduced levels of H2AK119ub1 found in cells derived from the affected individual. In contrast, RING1A protein levels were unaffected suggesting that the observed phenotype was a result of a change in RING1A function rather than its abundance. Engineering this mutation in SPAT-3, the RING1 orthologue in *C. elegans*, resulted in reduced ubiquitination and abnormal neuronal cell migration and axon guidance patterns in agreement with previous observations [113,114]. Recent work in *C. elegans* has also identified a role for SPAT-3 in neuronal cell fate specification in a manner that protects the differentiation programme from environmental stress [115].

The overwhelming burden of evidence suggests that RING1A is functionally subordinate to RING1B in mammals; however, abrogated RING1A function leads to reduced H2AK119ub1 and disordered neurodevelopment. This observation suggests that the RING1 paralogues are not entirely redundant, hinting at unappreciated context specific functionality for RING1A during brain development.

### 5.2. PHC1

As previously discussed, PHC proteins are important constituents of cPRC1 and play a central role in its capacity to coordinate both local and distant chromosomal interactions [37,39,42]. Disruption of PHC1 gives rise to posterior skeletal and neural crest defects, including parathyroid and thymic hypoplasia and cardiac anomalies that often result in perinatal lethality [116,117,118,119].

In 2013, Awad and colleagues identified an individual bearing a novel missense mutation (p.L992F) in the SAM domain of PHC1 that was implicated in primary microcephaly pathogenesis [120]. This mutation led to PHC1 degradation and reduced levels of H2AK119ub1. Comet assays combined with gene expression analysis revealed impairment in DNA damage mechanisms and defective cell cycle progression, the latter of which is likely connected to the observed upregulation of Geminin; an important regulator of DNA replication progression. These effects were phenocopied upon PHC1 depletion, and rescued following PHC1 overexpression, supporting a direct role for PHC1 deficiency in the observed molecular and cellular deficits [120]. Whilst the L992F mutation causes no net change in charge, the exchange of an aliphatic for an aromatic residue may interfere with the ability of PHC1 to form head-to-tail interactions and consequently PRC1-dependedent higher order chromosomal interactions [37,42]. However, the mutation also results in reduced PHC1 levels and lowered H2AK119ub1, thus, it is impossible, without further experimental interrogation, to determine if structural or enzymatic functions are the primary drivers of the observed pathophysiology.

### 5.3. BCORL1

One of the most prominent non-canonical PRC1 subunits is BCL6 corepressor-like 1 (BCORL1). BCORL1 is linked to PRC1.1’s enzymatic core through PCGF1 and is thought to repress transcription through interactions with C-terminal binding proteins (CtBPs) and Class II histone deacetylases (HDACs) [64,121]. BCORL1 is ubiquitously expressed, although levels vary, with high expression in testis and prostate, and relatively lower levels in other tissues including the brain. Pathogenic hemizygous variants in *BCORL1* underlie the rare X-linked recessive NDD—Shukla-Vernon syndrome (SHUVER) [122]. SHUVER is characterised by varying levels of ID, delayed acquisition of developmental milestones, ASD and dysmorphic physical features [122,123]. In addition, affected individuals present with abnormal EEG recordings, cerebellar atrophy and seizures with varying penetrance [122,123,124].

None of the known pathogenic BCORL1 variants impair protein domains that are required for gene repression, including both the C-terminal binding protein domain (CBD) and PCGF Ub-like fold discriminator (PUFD) domain [122,124]. Consequently, the molecular aetiology of SHUVER, and the contribution of altered PRC1.1-function remain unknown. It is possible that pathogenic variants lead to abnormal cross-talk between BCORL1 and HDACs. HDAC4 and 5 are highly expressed in the hippocampus and cerebellum; brain regions that are associated with memory formation and motor function respectively [122]. BCORL1 mutations could therefore perturb interaction with HDACs leading to impaired cortical migration, neuronal differentiation, maturation and cerebellar development. Further investigation is required to determine the underlying mechanism of Shukla–Vernon syndrome and the contribution of altered histone acetylation and/or ubiquitination levels.

### 5.4. AUTS2

A second ncPRC1 subunit implicated in neurodevelopmental disorders is autism susceptibility candidate 2 (AUTS2). AUTS2 is expressed at high levels throughout the brain during early development and is linked to PRC1.3 and PRC1.5 through interaction with PCGF3 and PCGF5 respectively [24,125,126,127]. While the precise gene regulatory role of AUTS2 is not well understood, recent work has shed light on its function in the developing brain, where it is associated with the promoter and distal enhancer regions of active genes, including the neurodevelopmental regulator neurexin 1 (NRXN1) [128]. Consistent with this observation, and contrary to the canonical view of PRC1 as a repressor, AUTS2 appears to have a role in transcriptional activation via recruitment of the co-factors casein kinase 2 (CK2) and P300 [129]. The AUTS2:PRC1.5 complex has impaired E3 ubiquitin ligase activity due to CK2-mediated phosphorylation of RING1B at S168 [129]. These findings suggest that AUTS2 is simultaneously associated with P300 mediated deposition of histone acetylation, an active histone modification, and a less catalytically active form of ncPRC1. Furthermore, recent work has shown that AUTS2:PRC1.3 is required for neuronal progenitor differentiation [130]. This suggests a context-specific role for AUTS2 in gene activation during neural lineage commitment that is dependent on nuclear respiratory factor 1 (NRF1) recruitment to the genome and the presence of P300 and PCGF3 [130,131]. Considering that RING1B occupies thousands of genomic locations in NPCs that lack PRC2-deposited H3K27me3, these may represent sites of AUTS2:PRC1-mediated gene activation [27].

AUTS2 was originally linked to neurodevelopmental disorders in a study that described a pathogenic mutation found in a pair of monozygotic twins with ASD [125]. Since then, “AUTS2 syndrome” has been used to describe a wide range of clinically heterogeneous phenotypes in more than 50 unrelated patients with distinct alterations of the AUTS2 gene including genomic rearrangements, copy number variations, deletions and single nucleotide variants [132,133,134,135,136,137]. Despite this variability in the underlying genetic causes, individuals with AUTS2 syndrome generally present with microcephaly, behavioural difficulties and dysmorphic features.

Experimental exploration into the mechanism of this family of disorders has shown that AUTS2 deficiency leads to defects in neuronal development, neurite formation, neural migration and perturbed synaptic function; characteristics which correspond with AUTS2 disruption in humans [24,138,139,140,141]. The *AUTS2* gene encodes two alternative protein isoforms that are involved in a range of physiological roles in neural development and gene regulation [131,138]. The long AUTS2 isoform is expressed in mESCs, which is replaced by a short isoform upon neural differentiation [131]. Despite this, knockdown experiments in mice found that full-length AUTS2 located in the cytoplasm of developing neurons acts as an important regulator of neural migration and excitatory/inhibitory synapse balance [138]. In humans, it has been suggested that inherited small in-frame 5′ deletions result in a mild clinical phenotype, whereas de novo mutations in the highly conserved, albeit shorter 3′ transcript cause haploinsufficiency and a more severe phenotype [133]. This suggests a correlation between genotype and clinical severity, which may be related to AUTS2 isoform-specific functions, although this notion is not entirely consistent across the literature [133,142,143]. It is interesting to consider that the shorter nuclear only form of AUTS2 is thought to be involved in gene activation, which implicates perturbed function of AUTS2:PRC1 as a potential driver of more severe phenotype emergence. Another interactor in this pathway is WDR68, which has recently been found to be critical for neuronal differentiation; WDR68 is not only a key component of PRC1-AUTS2 interaction but also required for AUTS2-mediated transcription activation [144].

## 6. Disordered PR-DUB Function in Rare Congenital NDDs

Disruption of PR-DUB function is implicated in multiple pathological contexts, including malignant cancers and neurodevelopmental conditions. In addition to its roles in development, PR-DUB function in DNA repair, and indeed, mutations of PR-DUB subunits are frequently seen in melanomas and mesotheliomas [145,146,147]. This section will focus on the NDDs associated with mutations in the PR-DUB subunits ASXL1-3 and MBD5.

### 6.1. ASXL Paralogues and Disordered Brain Development

Humans and mice encode three homologues of *Drosophila Asx*–*ASXL1*, *2* and *3*, the products of which form mutually exclusive heterodimers with BAP1 to establish the catalytic core of the PR-DUB complex [25,27,148,149,150,151]. In both humans and mice, the ASXLs share a conserved domain structure with *Drosophila* Asx, although the overall sequence has diverged [150,152]. Consistent with this domain level conservation, there is some functional redundancy between the paralogues, with single knockouts impacting little on molecular and cellular processes relative to the impaired cell proliferation, altered gene expression and elevated H2AK119ub1 levels observed in Asxl1-3 triple knockout mESCs [85]. Despite this functional overlap, other studies have found evidence for individual roles for each of the ASXLs. As tumour suppressors, disruption of each of the ASXLs is associated with distinct cancers [152,153]. In adipogenesis, ASXL2 upregulates the PPARγ pathway, leading to the development of adipocytes, whereas ASXL1 downregulates the same pathway [154]. Experimental knockdown of asxl3 in Xenopus embryos leads to a reduction in the number of primary neurons [155]. Interestingly, cells deficient for BAP1 show an overlapping yet distinct gene expression profile to that of cells lacking ASXL1, 2 and 3, suggesting additional, BAP1- and/or ASXL-independent function [85].

Some of these differences could be attributed to context specific expression rather than functional differences; indeed the ASXLs have distinct expression profiles during mammalian development. In humans, *ASXL1* and *2* are expressed during embryogenesis; however whilst *ASXL1* transcript levels remain high in the forebrain into adulthood, *ASXL2* levels decrease in most tissues perinatally [96]. In contrast, *ASXL3/Asxl3* expression levels are low in neural progenitors but high in post-mitotic neurons in both humans and mice [156,157]. At the protein level, ASXL2 is the predominant ASXL family member to associate with the PR-DUB complex in both mESCs and NPCs [27].

De novo mutations in *ASXL1*, *2* and *3* cause Bohring–Opitz (BOS; [158,159]), Shashi–Pena (SPS; [160]) and Bainbridge–Ropers (BRPS; [161]) syndromes, respectively. These rare congenital neurodevelopmental conditions present with distinct yet overlapping physical, cognitive and behavioural characteristics. All three syndromes typically show ID and atypical craniofacial formation. Some characteristics like BOS posture in ASXL1-deficiency or glabellar nevus flammeus (a red birthmark on the forehead, directly above the nose) are seen in BOS and SPS but not BRPS (Key features of BOS, SPS and BRPS are summarised in Table 1) [158,160,161,162,163]. The commonalities between each syndrome fit with the idea that ASXLs are partially redundant, yet dissecting out the basis of divergent phenotypes is harder to address. Absolute ASXL abundance, context-specific expression and functional specificity will all contribute to the unique presentations of each syndrome.

Of particular interest is the divergence in head size between the syndromes, where macrocephaly is frequently observed in SPS and microcephaly is observed in BOS and some BRPS patients (Table 1; [159,165]). Given the known role of polycomb proteins in the regulation of stem cells and differentiation, and the specific contribution of PRC1 functions during neurogenesis, it is easy to hypothesise how abnormal control of H2AK119ub1 levels could lead to inappropriate balance between NSC expansion and differentiation [106,107,108,112]. However, the stark contrast between these phenotypes highlights the importance of understanding the significance of altered levels and distribution of H2AK119ub1 in different neural cell types and the consequence of this on brain growth and function.

### 6.2. MBD5 and MBD5-Associated Neurodevelopmental Disorder

MBD5 is a member of the methyl-CpG-binding domain (MBD) family of transcriptional co-regulators, and interacts with the PR-DUB complex in a mutually exclusive manner to its paralogue, MBD6 [25,27,87,167]. In humans, the *MBD5* gene is located on chromosome 2q23.1 and encodes two distinct protein isoforms; the longer primary isoform is highly expressed in brain and testis, and a second shorter isoform is highly expressed in ovaries. Both isoforms possess the characteristic MBD at their N-terminal, however, this domain does not bind methylated DNA, but is instead important for interactions with the PR-DUB complex [87,168,169]. The long isoform also possesses a C-terminal proline-tryptophan-tryptophan-proline (PWWP) domain that acts in concert with the MBD to localise MBD5 to chromatin [168]. In line with recent observations relating to PR-DUB function, reporter assays demonstrate that MBD5 can act as a transcriptional activator [85,86,103,170]. Mice deficient for MBD5, show impaired postnatal growth and glucose homeostasis and gene misregulation, particularly in the cerebral cortex [171,172].

The relative proportion of MBD5 that associates with BAP1 (PR-DUB) is higher in differentiated NPCs relative to mESCs [27]. Coupled with the observation that brain specific MBD5 deficiency in mice phenocopies the whole animal knockout, suggests that MBD5 is primarily important for brain development [171]. Consistent with this notion, alterations in the expression of MBD5 gene causes a rare NDD termed MBD5-associated neurodevelopmental disorder (MAND), a general term that encompases 2q23.1 deletion syndrome, 2q23.1 duplication syndrome and MBD5 heterozygous variants (reviewed in [173]). The majority (~84%) of the phenotypes of 2q23.1 deletion syndrome are caused by haploinsufficiency of MBD5, and these commonly include ID, developmental delay, severe speech impairment, seizures, motor delay, sleep disturbances, autistic behaviours, microcephaly and subtle dysmorphic features [174,175,176]. However, one study suggested that the full repertoire of phenotypes is caused not only by reduced MBD5 levels, but also by the loss of other genes within the deleted portion of the chromosome [177]. Indeed, MBD5-specific disruptions do not present with microcephaly and sleep disturbances in contrast to larger 2q23.1 deletions [176]. Furthermore, 2q23.1 duplication syndrome has highly overlapping phenotypes with deletion syndrome, with the exception of microcephaly and seizures [178]. 

These observations suggest that proper dosage of MBD5 is vital for normal PR-DUB function and consequently for neurodevelopment, but that some aspects of MAND are specific to the nature of the underlying causal genetic lesion. Even though it is known that MBD5 is necessary, its function and biochemical properties are poorly understood. Further investigation is required to understand how improper MBD5 dosage impacts on PR-DUB function, H2AK119ub1 levels and the cellular and developmental characteristics of MAND disorders.

## 7. Discussion and Perspectives

Genetic lesions that impair the polycomb system, one of the paradigms of epigenetic regulation, are a prominent cause of a range of NDDs. In this review, we have explored the impact of perturbations in two of these complexes—PRC1 and PR-DUB whose opposing actions control the deposition and abundance of the H2AK119ub1 mark.

### 7.1. H2AK119ub1 and the Mechanism of Transcriptional Repression

Protein ubiquitination is used as a control mechanism for a wide array of cellular processes, the mechanics of which relate, in part, to the linkage chemistry of the underlying ubiquitin conjugate [179]. With respect to PRC1 function, modification of H2A (and H2AZ/H2Av) is exclusively monomeric, however whilst K119 is the favoured target of RING1A/B (K120 in H2AZ), additional proximal lysines can also be modified, albeit at lower frequency (K118 in H2A; K121 and K125 in H2AZ) [8,9,81,180,181,182,183,184]. Structural analysis suggests that modification is restricted to monoubiquitination due to the constrained conformation of the chromatin-E2:E3 interface and steric hindrance with the monomeric ubiquitin product [180,182]. Current experimental evidence does not however exclude the possibility that both H2A copies are simultaneously ubiquitylated within a single nucleosome. PRC1 and H2A/H2AZ monoubiquitination are thought to be primarily repressive, however the identification of dually modified H2AZ bearing both ubiquitylation and acetylation (acH2AZub1) provides an interesting parallel to bivalent nucleosomes (coexistence of H3K4me3 and H3K27me3 within a single nucleosome) [181,185,186].

To determine why perturbations in H2AK119ub1 levels lead to neuropathologies, we must understand its contribution to gene regulation. PRC1 is a multi-functional family of complexes; however, recent investigations have provided conclusive evidence that H2AK119ub1 is central to PRC1-mediated gene repression [75,78,187]. Indeed both ablation of PRC1 catalytic activity and removal of the ncPRC1 complexes primarily responsible for H2AK119ub1 deposition lead to extensive target gene upregulation [54,75,78]. Then how does this occur at a molecular level? There are two prevailing models: (1) ubiquitin-mediated effector binding and (2) direct transcriptional inhibition. In the recruitment model, the repressive capacity of H2AK119ub1 is mediated by reader proteins that elicit the repressive effect. The best-characterised example of this is the recruitment of polycomb complex components (e.g., RYBP, JARID2 and AEBP2) that act to enhance reciprocal polycomb targeting onto chromatin [10,12,21,73,74,188]. In addition, the novel H2AK119ub1 binder—remodeling and spacing factor 1 (RSF1), has been proposed to mediate transcriptional repression [189]. In the direct repression model, it is suggested that H2AK119ub1 directly antagonises transcription, either through steric hindrance, or through inhibition of the transcriptional apparatus. Polycomb targets have been shown to give rise to short abortive transcripts associated with stalled transcription, and acute loss of PRC1 leads to rapid association of RNA polymerase II (RNAPII) and an increased frequency of transcriptional bursting [82,190,191]. However, these two mechanisms are not mutually exclusive and neither fully explains the observed dynamics of repression. Furthermore, two recent studies have identified a novel H2AK119ub1 binding domain located in an isoform of the de novo methyltransferase DNMT3A1. This domain contributes to context specific DNMT3A1 targeting, including in the developing cerebral cortex, and provides an unanticipated connection between the polycomb and DNA methylation systems [192,193]. Further biophysical assessment is required to elucidate the molecular mechanism of H2AK119ub1 in transcriptional repression.

### 7.2. Perturbed H2AK119ub1 Balance as a Convergent NDD Aetiology

Despite possessing counteracting functions, mutations in PRC1 and PR-DUB give rise to phenotypically related neurodevelopmental disorders, suggesting that an ‘optimal’ level of H2AK119ub1 is required for healthy brain development [120,122,124,133,159,160,161,163,164,165,173,174,177]. This Goldilocks scenario indicates that an appreciable gain or loss of H2AK119ub1 levels leads to pathological changes in gene expression. Recent work, primarily performed in mESCs, has provided critical insights into how this may operate at a molecular level [85,86,103,104,105]. PRC1 targets ubiquitination to specific gene loci through direct recruitment and robust feedback mechanisms, however, PRC1.3 and PRC1.5 also deposit low levels of H2AK119ub1 throughout the genome [54,105]. In contrast, BAP1/PR-DUB targets actively transcribed regions, but also operates to counteract pervasive global ubiquitination [85,86,103,104,105]. The combined impact of these two activities is to ensure that H2AK119ub1 accumulates predominantly at appropriate target genes [85,103,105]. Given that ubiquitination is a highly abundant modification that operates, at least in part, through a limited pool of reader proteins, it is clear why the coordinated effort of PRC1 and PR-DUB to set the location and abundance of H2AK119ub1 is required to maintain robust and targeted gene repression (Figure 3) [49,54,105]. Interestingly, recent work points towards a contrasting, dynamic interplay between PRC1 and PR-DUB during *Drosophila* development [194]. Whether this represents a functional divergence between mammals and flies is yet to be determined. There are equivalent observations in experiments investigating the relationship between the DNA methylation and polycomb systems. Global loss of DNA methylation leads to a marked re-distribution of polycomb complexes, altered chromatin architecture and target gene de-repression [195,196,197]. This fits with the notion that extensive cross-talk between chromatin modifications controls their spatial and quantitative distribution to establish context specific gene expression patterns.

There is currently insufficient evidence to determine if dysfunction of individual NDD-associated PRC1 and PR-DUB components contribute to a shared pathological gene expression signature in neural cell types. However, mutations in RING1A and ASXL1-3 would be predicted to directly alter H2AK119ub1 levels and mutations in AUTS2, BCORL1, PHC1 and MBD5 to alter the distribution and occupancy of their respective complexes. It is also interesting to note that both reduced and increased levels of the chromatin proteins MBD5 and MeCP2 lead to phenotypically related disorders [173,177,198,199]. This suggests that the levels of both chromatin readers and their associated modifications are finely tuned to establish appropriate gene expression patterns. However, it is important to remember that these complexes are multi-functional and that a subset of the subunits have additional, polycomb-independent functions. Further investigation with appropriate transgenic and cellular models is required to determine if the PRC1 and PR-DUB mutations converge upon a common molecular mechanism during neurodevelopment.

### 7.3. Non-Canonical Functions of PRC1 and PR-DUB in the Brain

It is important that we remain open to alternative functions of the polycomb system; both in terms of context and mechanism of action. The largest body of evidence relating to the genetics, underlying molecular basis and phenotype of any PRC1-associated NDD is that of AUTS2 syndrome [129,130,134,136,137]. In this case, the emerging view is that the characteristics of the syndrome are the sum of functions that are both dependent and independent of ncPRC1 [129]. In this case, AUTS2 and its associated subunits dampen the catalytic activity of PRC1.3 and 1.5, major contributors to the H2AK119ub1 pool in mESCs, and repurpose them for transcriptional activation [24,54,105,129,144]. Strikingly, the context-specific inclusion of these subunits has been proposed to subvert these complexes to a function that is diametrically opposite to the conventional function of PRC1. It should be noted however, that inhibition of RING1B by CK2 has only been demonstrated in vitro, and interaction between PRC1.3/1.5 and CK2 in mESCs does not lead to reduced H2AK119ub1 deposition [27,54,129]. Further experiments are, therefore, required to resolve these seemingly discrepant observations.

RING1A and PCGF4 are implicated in the ubiquitination of chromatin-associated proteins with a speculated role in mitotic bookmarking [200,201]. Furthermore, RING1B and BAP1 (PR-DUB) can autoubiquitylate and excise ubiquitin molecules from non-chromatin targets respectively [93,202,203,204,205]. In addition, we justifiably focus much of our attention on the role of polycomb proteins during development. However, work from the Schafer lab has demonstrated an important role for PRC2 in silencing a deleterious gene expression programme to protect against neurodegeneration in adult mouse neurons [206]. Combined with the fact that many PRC1 and PR-DUB subunits are expressed throughout the adult brain, this obviates the need to understand polycomb-mediated gene regulation in a broader context than we do at present. Additional targets and cellular processes are likely impacted by such non-canonical activities, and these will need to be identified in order to provide a complete basis on which to interpret the modus operandi of NDD-causal mutations.

### 7.4. Concluding Remarks

The commonalities between the NDDs discussed here are suggestive of a unifying disease mechanism. However, context-specific expression and function, variable phenotypic penetrance, unappreciated functionality and complex genetic underpinnings make determining such relationships challenging. With respect to this last point, it is interesting to note that whilst the vast majority of the known NDD-associated mutations occur de novo in the affected individuals, examples of inherited mutations associated with milder phenotypes exist for both ASXL3 and AUTS2 [133,207]. In these cases, the intergenerational penetrance and severity of the associated disorders is variable, likely due to genetic variations between family members. Whilst this appears to be yet another roadblock in the pursuit of mechanistic understanding, a range of distinguishable genotype–phenotype relationships will likely provide critical insights into disease mechanisms. Leveraging these genetic insights, in combination with appropriate molecular, cellular and developmental models, will allow us to decipher the underlying mechanisms of NDDs caused by PRC1 and PR-DUB dysfunction.

## Figures and Tables

**Table 1:** Key clinical features of BOS, SPS and BRPS.

Characteristic	ASXL1/BOS	ASXL2/SPS	ASXL3/BRPS
hypertelerism (wide-set eyes)	In some ([158,159])	Yes ([160])	Yes ([164])
upslanting palprebral fissures (opening between upper and lower eyelid)	Yes ([158]), In some ([159])	-	In some ([164])
downslanting palpebral fissures	-	-	Yes ([162,164])
glabellar nevus flammeus (a red or pink birthmark above the bridge of the nose)	Yes ([158,165])	Yes ([160])	-
macrocephaly (enlarged head)	-	Yes ([160])	-
microcephaly (small head)	Yes ([159,165])	-	In some ([161,163,164])
trigonocephaly (pointed forehead)	Yes ([158,159])	-	-
Low birth weight	Yes ([158])	No ([160])	In some ([161,164])
Feeding difficulties	Yes ([158,159,165])	Yes, at birth but not persistent ([160])	Yes ([161,163,164])
Hypotonia	Yes ([159,165])	Yes ([160])	Yes ([161,162,163,164])
Intellectual disability	Yes ([165])	Varying severity ([160])	Yes ([161,162,163,164,166])
Autism or Autistic features	-	Some autistic behaviours ([160])	Diagnosed or suspected in many ([166])
Seizures	Yes ([158,165])	In some ([160])	In some ([162,163,164])
BOS posture	Yes ([158,159])	-	-
